# Culturally sensitive approaches in digital mental health research: lessons learned from co-developing the core mental health dataset

**DOI:** 10.3389/fdgth.2026.1812227

**Published:** 2026-07-22

**Authors:** Heidi Tranter, Madeleine France Ratcliffe, Thomas Price, Auden Edwardes, Pauline Whelan, Kathryn M. Abel

**Affiliations:** 1 GM.Digital Research Unit, Greater Manchester Mental Health NHS Foundation Trust, Manchester, United Kingdom; 2 Centre for Women’s Mental Health, Division of Psychology & Mental Health, Faculty of Biology, Medicine & Health, University of Manchester, Manchester, United Kingdom; 3 Department of Sport and Exercise Science, Manchester Metropolitan University, Manchester, United Kingdom; 4 Division of Informatics, Imaging and Data Science, School of Health Sciences, Faculty of Biology, Medicine and Health, University of Manchester, Manchester, Greater Manchester, United Kingdom

**Keywords:** co-development, cultural sensitivity, mental health, mental health data, PPIE

## Abstract

Cultural sensitivity is central to ethical and valid mental health research, yet we observed it is often insufficiently embedded in digital research tools. Drawing on qualitative work conducted during the co-development of the Core Mental Health Dataset (CMHDS), this commentary reflects on the practical challenges we encountered and the considerations which shaped our approach to culturally sensitive data collection in digital mental health research. We synthesise insights from a lived experience advisory group, recognising how our interpretations were informed through dialogue with participants, focusing on mental health terminology, perceptions of mental health and ethical considerations in research. This paper shares lessons learned from co-developing the CMHDS and advocates for culturally sensitive digital approaches to improve acceptability, inclusivity, and data quality. We emphasises the requirement of sustained co-production and ongoing reflexive design practices in addressing cultural sensitivity in digital mental health research.

## Introduction

Cultural sensitivity is essential for digital mental health research, our work on the CMHDS prompted us to reflect on how this is conceptualised and operationalised in practice. It involves recognition and awareness of how factors such as ethnicity, religion, gender and socioeconomic status influence a person's perception of their health and illness, as well as the behaviours that might ensue from those perceptions. Cultural awareness is increasingly understood to be important not only in fostering better communication, better patient-centred care and better outcomes, but also in addressing inequalities resulting from culturally insensitive practices and poor patient engagement. Through our work, we became aware of the tension between standardised data collection approaches and the need for culturally responsive design. Adopting a culturally sensitive approach within digital mental health research can improve patient satisfaction and enhance the skills and knowledge of healthcare providers, ensuring outputs are both relevant and accessible to the target population (1).

The American Psychological Association (APA) guidelines (2), emphasise the ethical responsibility of researchers to conduct culturally appropriate and ecologically embedded research. Despite this, we recognise that health research may inadvertently overlook the needs of diverse and vulnerable populations, potentially exacerbating health disparities (1, 3). A range of challenges undermine the validity and ethical integrity of studies involving diverse populations (3–5). Digital health approaches have the potential to enhance accessibility, personalisation, and inclusivity in research (6) and to become an essential tool in developing strategies that enhance the quality of research findings, while also preventing further marginalisation of diverse communities (7). This commentary reflects on our lessons learned through the co-development of the Core Mental Health Dataset (CMHDS). In co-developing the CMHDS, we identified the challenges of implementing culturally sensitive approaches in digital mental health research where stigma and unrepresentative sampling risk findings not being applicable across diverse populations.

## Methods

Our National Institute for Health and Care Research (NIHR)-funded project (NIHR201104) co-developed the CMHDS to embed routine collection of mental health data in physical health research. The CMHDS consists of two psychometrically robust and globally validated self-report questionnaires for common mental health symptoms (PHQ-8 (8) and GAD-7 (9)), alongside novel questionnaires about mental health diagnoses, support sought and the experience of childhood or other forms of trauma. While these measures are widely used, we became aware of assumptions about the use of standardised measures, prompting us to consider their cultural appropriateness. Consent is stepped, allowing participants to complete some, or all questions. The aim of the CMHDS is to safely, securely, acceptably, and easily collect mental health information from participants in physical health research and thereby to grow understanding of the links between physical and mental health. The CMHDS was culturally adapted in 2023 through a separate project funded by the NIHR Applied Research Collaboration Greater Manchester. The cultural adaption of the CMHDS followed an iterative and agile design process (10) employed in previous cultural adaptation and co-development projects (11, 12). Throughout this process, we sought to remain attentive to how the assumptions and priorities of the study team could be shaping the methodological choices, recognising adaptation is not a neutral process.

We subsequently engaged the DATAMIND (13) (funded by the Medical Research Council and United Kingdom Mental Health Goals Programme) Lived Experience Advisory Group (LEAG) on two separate occasions across 2024 and 2025 to gather feedback on the culturally adapted CMHDS. Members of the LEAG (*N* = 8), were remunerated for their time according to NIHR guidelines. As we wanted to understand about cultural sensitivity in collecting information about mental health in people in physical health settings, all participants considered themselves service users or experts by experience with personal experience of interaction with health services for their mental health, but not necessarily for their physical health. All had a willingness to contribute insights to inform and shape the research. Members voluntarily shared a wide range of backgrounds, varying levels of research experience, diverse knowledge and perspectives on the secondary use of data, personal experiences with mental health conditions and services, as well as their protected characteristics. We recognise the feedback reflects the specific composition of this group and does not necessarily represent all cultural perspectives.

Members of the LEAG were asked to review the adapted CMHDS, and feedback during a focus group discussion. Following the same approach as previous research (11, 12), we captured data through notes and summaries rather than verbatim transcription. We acknowledge this approach involved some degree of interpretive decision-making from the study team. At the end of the discussion, the study team reflected the key changes to the CMHDS which could be made and explained why some of the changes discussed would not be included. This methodology emphasises the importance of information power (14), recognising depth and relevance of insight, rather than sample size alone, informed our approach.

## Mental health terminology

A key topic of the consultations was the diversity of cultural norms and values and the language used in the digital tool. Specifically, terms like “mental health problem” and “mental illness” were identified as potentially problematic, because their interpretation might differ widely between cultural contexts. We interpreted this feedback as reflecting concerns about the variability of meaning across cultural contexts and the potential for certain terms to reinforce stigma. The LEAG proposed the term “mental health condition” as a more neutral and culturally sensitive alternative. This prompted us to reflect on how language choices within the CMHDS could shape participant engagement and willingness to disclose information. We became increasingly aware that terminology is not simply descriptive but actively constructs understandings of mental health and influences interactions with research. It highlights the critical role that careful language selection plays in collecting valid and meaningful data. One review of 130 studies on mental health reported 24% used the term “mental health problems” (15) even though for some it may contribute to stigma and discourage help-seeking and reinforce misconceptions about the treatability of poor mental health (16, 17). Our experience reinforced the need for ongoing reflexivity in language selection, recognising no single term will be universally appropriate and that cultural meanings remain fluid and context-dependent.

## Perceptions of mental health

Different cultures have varied ways of understanding and expressing mental health. While mental ill health is stigmatised globally, stigma is more pronounced in certain communities (18). Qualitative findings highlight how culturally diverse populations may fail to talk about mental ill health and difficulties are not spoken about to “save face” or protect family reputation and honour (18). How individuals interpret and express their symptoms can also vary significantly across cultural contexts (19) with a diverse range of distress idioms utilised. We understood these insights as illustrating how cultural norms shape both the expression of distress and engagement with research. A qualitative study in Southeast England including 26 adults from Black and minority ethnic backgrounds identified personal and environmental factors that influenced access to mental health services (20). These included an inability to recognise and accept mental health conditions, a positive effect of social networks, male reluctance to discuss psychological distress and seek help, cultural identity, negative perception of, and social stigma against mental ill health, and financial factors (20).

How people access mental health support also varies across cultures. The cultural adaptation of the CMHDS included questions about religion and spirituality when asking about whether someone has received mental health support. Adaptations also included clear definitions of clinical diagnoses to enhance cultural sensitivity. For example, retaining diagnostic labels, such as “anorexia”, while adding plain-language descriptions was intended to bridge potential gaps in understanding, though we acknowledge this may not fully capture culturally specific conceptualisations of mental health. This reflects the research of Hall et al. (21) who suggest that pragmatic approaches focused on helping individuals cope with specific external problems, compared to managing a “personal” disease, can effectively “restore” face. Additionally, the LEAG pointed out that addiction-related questions were omitted from the CMHDS, and felt this information was necessary for assessment of mental health. It was clarified in the LEAG consultations that addiction questions would not be included in the CMHDS, but that this data could be collected separately as needed. The focus of the CMHDS is explicitly mental health data collection. This decision reflects pragmatic constraints but also illustrates how definitional choices shape what data are included or excluded.

## Ethical considerations in research

Respecting cultural values whilst adhering to ethical research standards can be difficult for researchers to navigate. Discussions with the LEAG included concerns that scaled questions in the digital tool might discourage meaningful responses, because they were too easy to ‘click through’ without engaging fully with the content. Free-text responses were suggested as a way to capture richer data while enhancing cultural sensitivity. We recognised the value of this insight but also reflected on the practical and ethical implications of incorporating free-text data. Whilst free-text responses may indeed enrich data capture, we should be mindful of collecting personally-identifiable details, a concern echoed in previous focus groups and in the literature (22). This emphasises the need for researchers to manage potentially identifiable disclosures and subsequent difficulties with governance and data integrity. The act of moderation raises issues relating to consent, participant trust and data authenticity. These considerations required us to make deliberate design decisions and to acknowledge the trade-offs involved.

The LEAG suggested the sensitive nature of mental health and trauma mean free text questions could trigger strong emotions in participants asked to reflect on past traumatic experiences (23). This identified a clear need for effective safeguarding when working with individuals who have experienced trauma. Individuals from ethnic minority groups often do not access formal mental health support (24). By collecting free-text data about experiences of mental health difficulties and access to support this may lead to participants feeling a lack of trust or worry about the safety of their data (25, 26). As a result of this, they may be less likely to complete the CMHDS. These considerations illustrate the care that needs to be taken in balancing data value with safeguarding participant well-being. In response, we developed an alternative version of the CMHDS incorporating optional free-text responses, allowing research teams to determine appropriateness based on context. This decision reflects an attempt to balance flexibility with ethical responsibility, while acknowledging that no single approach will be suitable in all settings.

## Conclusion

Through our work of co-developing the CMHDS, we understand digital mental health tools present a real opportunity to enhance access to better care. Implementing culturally sensitive recommendations in the development of these will be crucial to ensure their added value is realised and that data and outcomes emerging from them can be generalised across cultural divides. We acknowledge that our work is situated within specific methodological and contextual constraints, and that further research is needed to explore how culturally sensitive approaches can be embedded at scale. Our reflections emphasise the importance of balancing psychometric robustness with cultural responsiveness, recognising standardisation should not come at the expense of inclusivity. Digital tools may be particularly helpful in addressing language and capturing more culturally diverse perceptions of mental health. In the pursuit of culturally-sensitive and inclusive services, ethical concerns and participant trust are key to ensure a collaborative approach and mitigation of ethnocultural bias ultimately ensuring adaptations to research are inclusive and relevant to diverse populations. A balance should be maintained between collecting data using psychometrically valid measures whilst embedding culturally sensitive approaches. Digital mental health research will undoubtedly play an important role in widening and scaling access so the people who participate in research are likely to need the treatments derived from it. Digital mental health research will undoubtedly play an important role in widening and scaling access, therefore it is critical that the people who access treatment are reflected in the research that contributes to its development. We suggest sustained engagement with diverse communities, alongside reflexive design practices, is essential to support the development of equitable and culturally sensitive mental health research.

## Data Availability

The original contributions presented in the study are included in the article/Supplementary Material, further inquiries can be directed to the corresponding author.
